# On-Surface Synthesis of Hexabenzocoronenes via a Retro-Büchner Reaction of Heptaarylcycloheptatrienes

**DOI:** 10.1021/acs.jpclett.6c01675

**Published:** 2026-08-25

**Authors:** Shigeki Kawai, Haruka Tsuji, Donglin Li, Pranshu Pal, Oscar Custance, Toshikaze Kariyado, Yorie Ohno, Yohei Hattori, Thomas A. Hector, Claire Kammerer, Gwénaël Rapenne

**Affiliations:** † Center for Basic Research on Materials, National Institute for Materials Science, 1-2-1 Sengen, Tsukuba, Ibaraki 305-0047, Japan; ‡ Graduate School of Pure and Applied Sciences, University of Tsukuba, Tsukuba, Ibaraki 305-8571, Japan; § Department of Metallurgical and Materials Engineering, 195695 National Institute of Technology Srinagar, Hazratbal, Srinagar, Jammu and Kashmir 190006, India; ∥ Research Center for Materials Nanoarchitectonics (MANA), National Institute for Materials Science, 1-1 Namiki, Tsukuba, Ibaraki 305-0044, Japan; ⊥ Division of Materials Science, Nara Institute of Science and Technology, 8916-5 Takayama, Ikoma, Nara 630-0192, Japan; # Université de Toulouse, CNRS, CEMES, 29 Rue Marvig, 31055 Toulouse, France

## Abstract

On-surface synthesis has become a powerful technique for obtaining various nanostructures, mainly by linking small precursor molecules. In contrast, the cleavage of specific moieties from precursor molecules is also an important and promising strategy to increase the variety of products. Here, we report the on-surface synthesis of hexabenzocoronene (HBC) and its hexa-fluorinated analogue (6F-HBC) from the parent heptaphenylcycloheptatrienes, via the systematic extrusion of a phenyl-CH: carbenic fragment and a subsequent cyclodehydrogenation process on a Au(111) substrate. Their structures and electronic properties were investigated in-depth by low-temperature scanning tunneling microscopy with a CO-terminated tip. Annealing at 200 °C triggers the elimination of the phenylcarbenic groups from the cycloheptatriene core, resulting in the formation of both hexaphenylbenzene and its fluorinated analogue. Although the former was transformed into HBC via cyclodehydrogenation at 300 °C, the fluorinated one was thermally desorbed from the surface even at 250 °C. We found that depositing fluorinated heptaphenylcycloheptatriene onto the substrate kept at 300 °C successfully yielded 6F-HBC. The influence of fluorine substitution was identified through molecular assemblies and bond-resolved images as well as d*I*/d*V* measurements, all obtained using the same CO-terminated tip. Our findings demonstrate that the retro-Büchner reaction, corresponding to a ring contraction, is an effective strategy to enhance the variety of structures and electronic properties of on-surface synthesized products.

## Graphical abstract

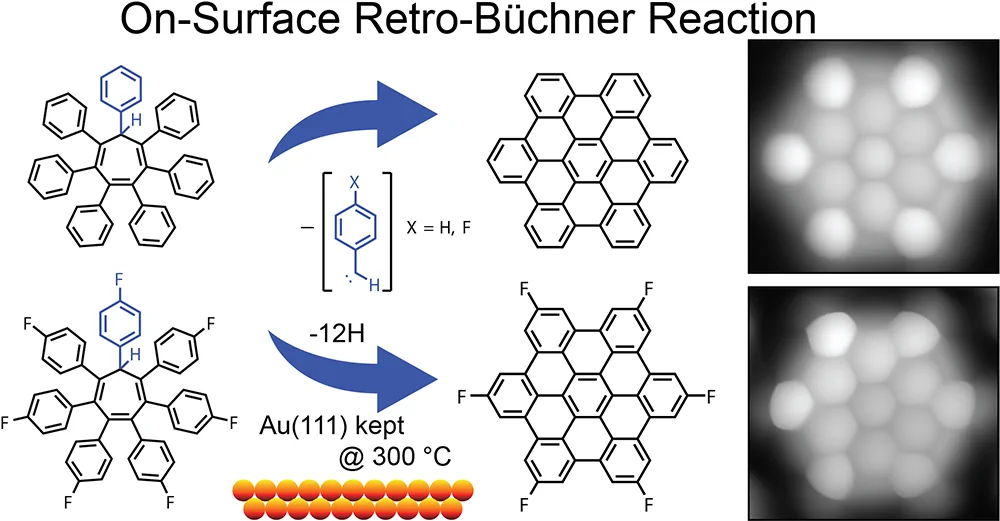

Cycloheptatriene derivatives are non-benzenoid, seven-membered cyclic hydrocarbons that have attracted sustained interest due to their unique structural and electronic properties. Unlike benzene, cycloheptatriene derivatives adopt a nonplanar conformation and contain a localized sp^3^-hybridized methylene group, which interrupts full conjugation across the ring.^1^ They are known to generate the stable cycloheptatrienylium cation^2^ (known as tropylium), an aromatic cationic counterpart, via hydride abstraction. In heptaarylcycloheptatrienes, the presence of seven aryl substituents not only stabilizes the cycloheptatriene framework but also significantly influences its conformational dynamics and electronic properties through steric congestion and conjugative effects. Cycloheptatriene derivatives are efficiently synthesized by the Büchner ring expansion reaction.^3,4^ In a first step, a carbene is generated and reacted with a benzenic ring to yield a cyclopropanated benzene ring, which rearranges through a ring expansion to give access to a wide variety of cycloheptatriene-based chemical structures.^5^

On-surface synthesis has emerged as a powerful bottom-up strategy for constructing atomically precise nanocarbon architectures.^6^ Designed precursors are typically synthesized through organic chemistry in solution and then deposited on surfaces under ultrahigh-vacuum conditions. Subsequent surface-assisted reactions yielded the desired products. Since the first successful debrominative homocoupling of porphyrins,^7^ the Ullmann-type reaction has been widely utilized. By incorporation of subsequent cyclodehydrogenation^8,9^ steps, graphene nanoribbons have been successfully synthesized on surfaces.^10^ To increase the variety of structures, it is of central importance to develop further on-surface reactions, such as Glaser couplings.^11^ In addition to coupling reactions that extend molecular structures, the dissociation of specific groups from precursors is also a key topic for modifying the molecular backbones. To this end, cleavages of C–C bonds, C–N bonds, CN groups, trimethylsilyl (TMS) groups, and methyl groups have been demonstrated.^12–21^ However, in contrast to the rich demonstration of coupling, the dissociation of certain groups has been less explored.

Here, we report the on-surface synthesis of HBC^22^ and 6F-HBC^23^ via the elimination of arylcarbenic fragments, followed by cyclodehydrogenation on Au(111) from heptaphenylcycloheptatriene (**1**) and hepta­(*p*-fluorophenyl)­cycloheptatriene (**4**), respectively (Scheme 1). While the non-fluorinated precursor was transformed into HBC (**3**) through hexaphenylbenzene (**2**) by stepwise thermal annealing at different temperatures, the fluorinated precursor underwent desorption at 250 °C after transformation into hexa­(*p*-fluorophenyl)­benzene (**5**). In contrast, depositing the precursors on a Au(111) substrate kept at 325 °C yielded both HBC (**3**) and 6F-HBC (**6**). We found that the fluorine substituents significantly induce electronic distortion in the outer phenyl rings and shift the level of the molecular positive/negative ion resonances (PIR/NIR), although their spatial distributions were almost identical.

**Scheme 1:**
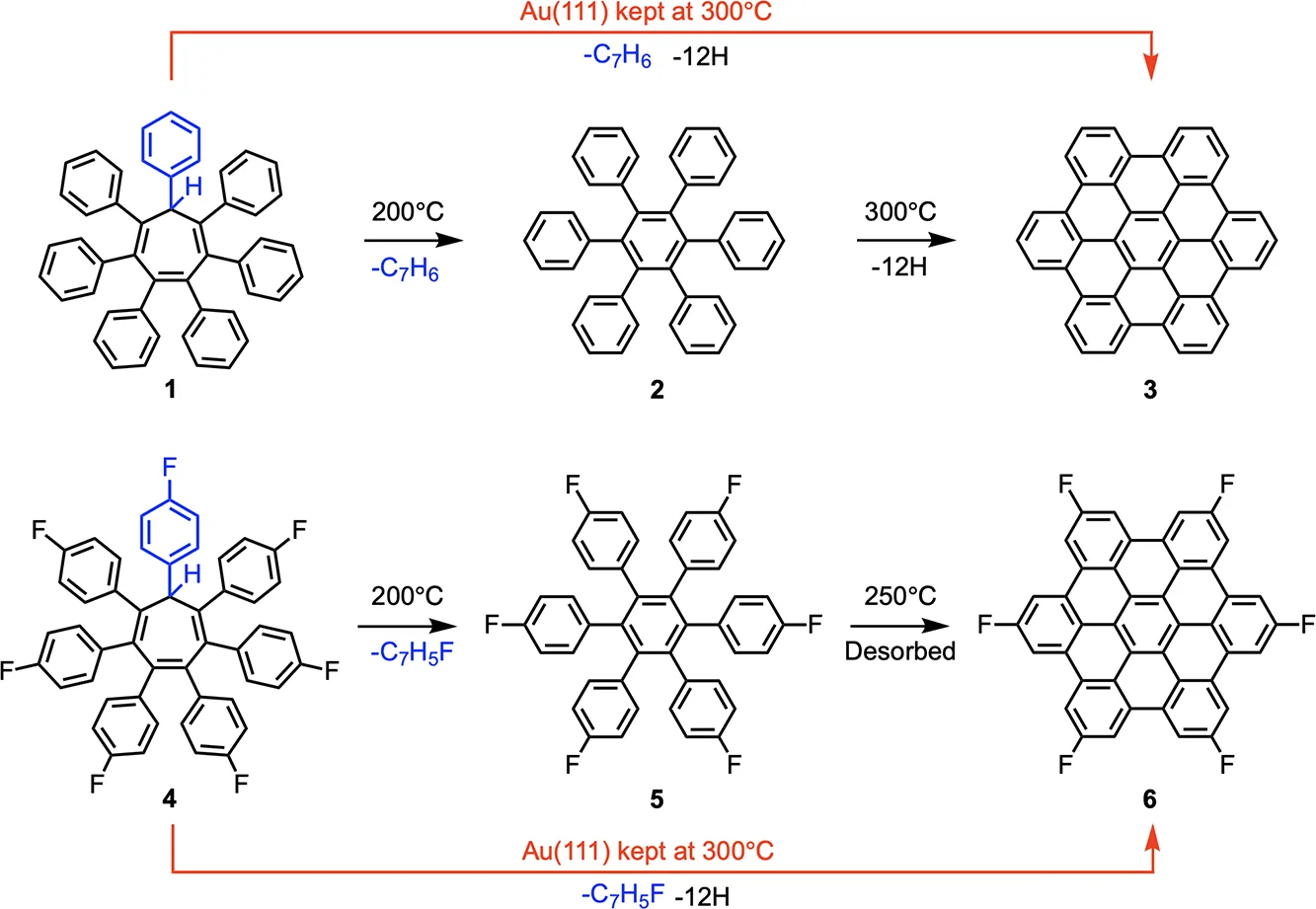
Synthetic Routes toward Hexabenzocoronene (HBC, **3**) and 2,5,8,11,14,17-Hexafluoro-hexa-*peri*-hexabenzocoronene (6F-HBC, **6**) via the Elimination of Phenylcarbenic Fragments and Subsequent Cyclodehydrogenation on Au(111) from Heptaphenylcycloheptatriene (**1**) and Hepta­(*p*-fluorophenyl)­cycloheptatriene (**4**), Respectively

Heptaphenylcycloheptatriene (**1**) was synthesized following a published procedure,^24^ while its fluorinated analogue, hepta­(*p*-fluorophenyl)­cycloheptatriene (**4**), was prepared in three steps from reported building blocks according to Scheme 2 (see the Supporting Information for more details).

**Scheme 2:**
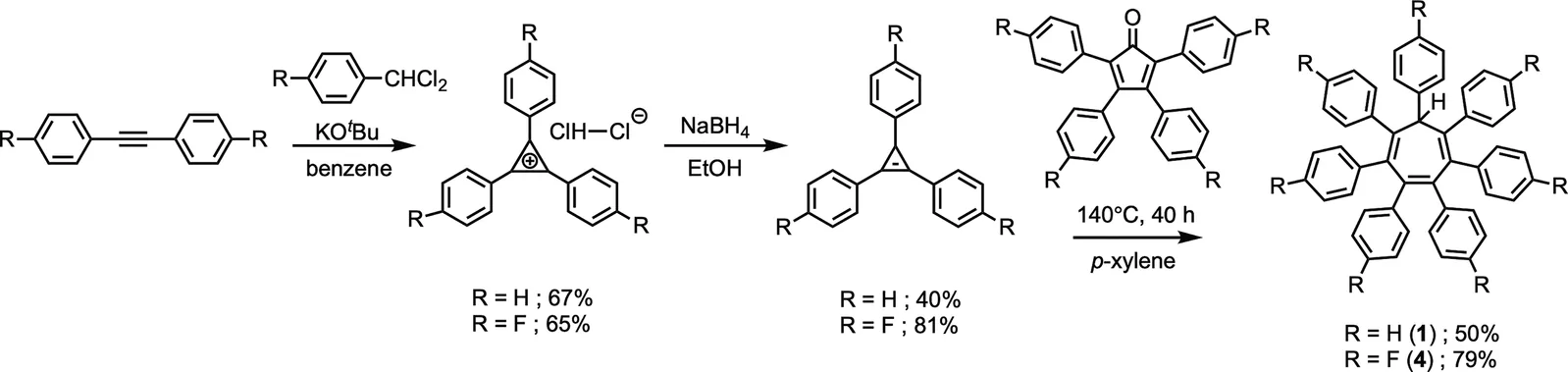
Synthetic Route to Prepare Hepta­(*p*-fluorophenyl)­cycloheptatriene (**4**)­^a^

a All details and characterization data can be found in the Supporting Information.

Upon depositing heptaphenylcycloheptatriene (**1**) on a Au(111) surface at room temperature, the surface was characterized by scanning tunneling microscopy (STM) under an ultrahigh-vacuum environment at 4.5 K. A ladder-like self-assembled structure with a spacing of approximately 3.2 nm was formed (Figure 1A). The longitudinal axis of the structure is rotated relative to the substrate lattice, and the angle between growth directions of the two structural domains was approximately 54°, which is inconsistent with the substrate symmetry. The longitudinal axis of the ladder-like structure was also slightly curved near the step, as indicated by blue broken lines. Imaging the sample was relatively challenging because the tip often accidentally picked up a molecule from the surface. Although the corrugated film obscures the small feature of the herringbone structure, it can still be observed onto the self-assembled film (Figure S11), indicating weak molecule–substrate interactions (physisorption). Figure 1B shows a close-up view of the self-assembled structure. No clear short-range periodicity was observed in the ladder-like assembly. Nevertheless, since precursor **1** adopts a corrugated conformation on the surface due to the seven tilted phenyl groups connected to the nonplanar cycloheptatriene core, the molecules are likely condensed via the phenyl–phenyl contact, which induces π–π and CH−π interactions.^25,26^ Owing to the flexibility of the core and the complexity of these interactions, our attempt to assign the molecular arrangement in the assembled structure was unsuccessful. Nonetheless, we deduce that the weak interaction between the corrugated molecule and the substrate renders the structure incommensurate with the surface lattice. A similar feature was observed in our previous study using molecules with a pentagonal core.^27^ After annealing the sample at 200 °C, the molecular film became flatter and the contrast associated with the herringbone reconstruction enhanced (Figure 1C). The close-up view (Figure 1D) shows that the molecules were self-assembled with the 3-fold symmetry. Each molecule exhibited six bright dots instead of seven, although the contrast in the STM topography varies significantly between dots and molecules. This observation is in agreement with the previous study on hexaphenylbenzene,^28^ indicating that a phenylcarbenic fragment Ph–CH: was eliminated from heptaphenylcycloheptatriene (**1**) upon annealing. Next, to synthesize HBC via cyclodehydrogenation of **1**, the sample was annealed at 300 °C.^28–31^ We found the presence of isolated flat molecules, while most molecules fused with each other (Figure 1E). Some brighter contrasts correspond to the partially planarized molecules. To investigate the structure, the apex of the STM tip was terminated with a CO molecule.^32,33^ The bond-resolved image unambiguously confirmed the synthesis of HBC (Figure 1F). Since the intermolecular interaction between HBCs on Au(111) results from repulsive C–H···H–C contacts, no self-assembled structure was observed at low coverage.^34^ Large structures were formed by random fusion between HBCs as well as their defects caused by further dissociation of phenyl groups (Figure S12).

**Figure 1:**
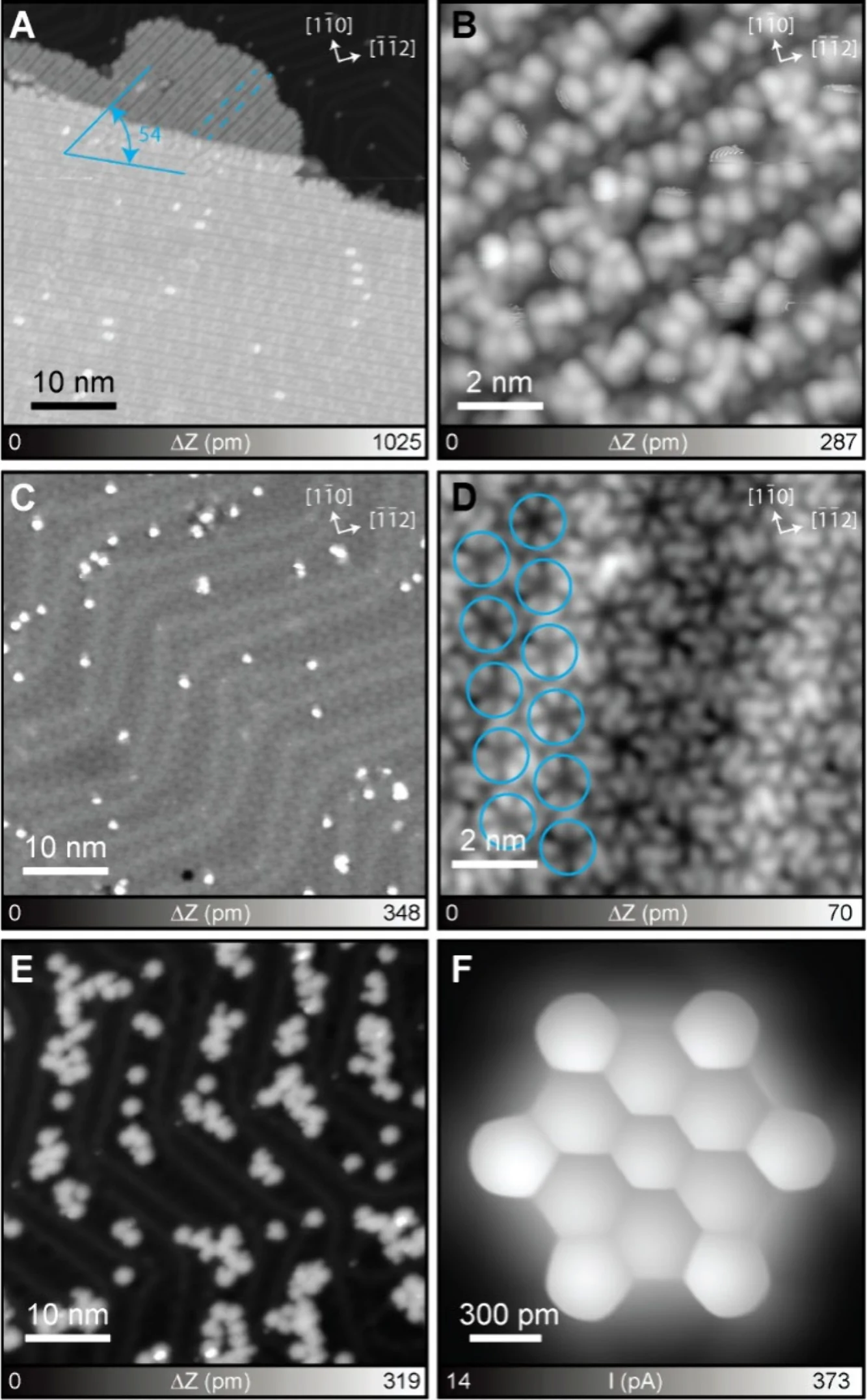
On-surface synthesis of HBC. (A) Large-scale STM topography of self-assembled **1** on Au(111) and (B) close-up view. (C) Large-scale STM image taken after annealing at 200 °C and (D) close-up view. Blue circles indicate individual molecules. (E) Large-scale STM topography taken after annealing at 300 °C. (F) Constant-height bond-resolved image of hexabenzocoronene, obtained using a CO-terminated tip. Scanning parameters: (A) *V* = 200 mV and *I* = 5 pA. (B) *V* = 200 mV and *I* = 20 pA. (C) *V* = 200 mV and *I* = 10 pA. (D) *V* = 200 mV and *I* = 5 pA. (E) *V* = 200 mV and *I* = 10 pA. (F) *V* = 10 mV.

Next, hepta­(*p*-fluorophenyl)­cycloheptatriene (**4**) was deposited on a clean Au(111) surface kept at room temperature. Self-assembled ring-like structures were formed, which are aligned along the [1 −1 0] substrate direction, as indicated by circles with a diameter of 2.5 nm (Figure 2A). Based on their size, these ring-like structures apparently consist of several molecules and a significant number of irregular structures were also observed. Figure 2B shows a close-up view of the molecular film. The ring-like structure is composed of three condensed protrusions at the center (green arrows), surrounded by six pairs of dimers, as indicated by blue arrows. However, the arrangement varies between individual ring-like structures; therefore, no clear periodicity was identified. Thus, similar to **1**, structural analysis of the molecular film was challenging. After annealing the sample at 200 °C, periodic self-assembled structures were observed, in which the molecules were perfectly aligned along the [1 −1 0] direction of the substrate (Figure 2C). The distinct appearance of the herringbone pattern onto the film indicates weak molecular–substrate interactions. The close-up view reveals that each molecule has six bright spots, suggesting that fluorinated phenylcarbene groups were dissociated to yield hexa­(*p*-fluorophenyl)­benzene (**5**) (Figure 2D). The tilt direction of the phenyl groups relative to the substrate varies, as indicated by blue and green arrows (Figure S13). To synthesize 6F-HBC, the sample was annealed at 250 °C. However, in contrast to the stepwise thermal synthesis of HBC from hexaphenylbenzene at 300 °C (see above), most of the molecules were desorbed from the surface, indicating that the fluorine substitution significantly weakens the molecule–substrate interactions. To avoid desorption of **5** before transforming to 6F-HBC, precursor **4** was directly deposited on a clean Au(111) substrate kept at 300 °C, and we obtained a self-assembled structure (Figure 2E). In contrast to HBC, the absence of isolated molecules indicates that intermolecular interactions are attractive. To resolve the inner structure, the tip apex was terminated with a CO molecule. The bond-resolved image confirms the synthesis of 6F-HBC (Figure 2F). We also observed that side products were synthesized through the elimination of an additional *p*-fluorophenyl fragment or the formation of an additional hexagonal ring (Figure S14). However, in contrast to HBC, the amount of defects was relatively small, indicating that the thermal stability of 6F-HBC is greater than that of HBC (Figure S15). Characterization of the self-assembled structure using bond-resolved images confirms that 6F-HBCs were condensed by C–F···H–C hydrogen bonding.^35^

**Figure 2:**
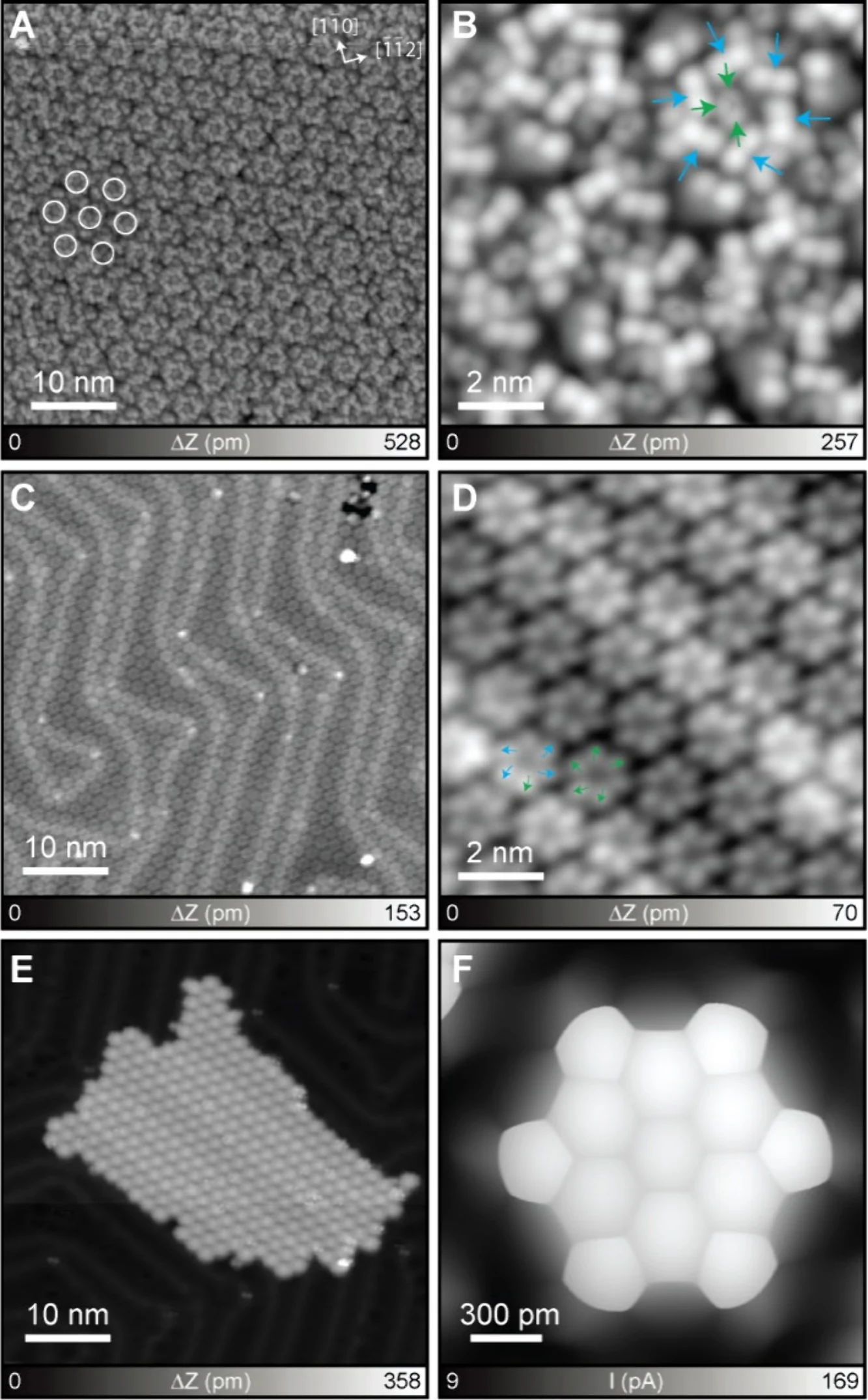
On-surface synthesis of 6F-HBC. (A) Large-scale STM topography of a self-assembly of **4** on Au(111) and (B) close-up view. Blue and green arrows denote three productions at the center and six pairs of dimers in the typical ring-like structure, respectively. (C) Large-scale STM image taken after annealing at 200 °C and (D) close-up view. Blue and green arrows denote tilted phenyl groups in anti-clockwise and clockwise directions, respectively. (E) Large-scale STM topography taken after depositing **4** on a clean Au(111) substrate kept at 300 °C. (F) Constant-height bond-resolved image of 6F-HBC, obtained using a CO-terminated tip. Scanning parameters: (A and B) *V* = 500 mV and *I* = 5 pA. (C) *V* = 200 mV and *I* = 5 pA. (D) *V* = 200 mV and *I* = 50 pA. (E) *V* = 200 mV and *I* = 10 pA. (F) *V* = 5 mV.

To investigate the structural and electronic properties of both HBC and 6F-HBC using the same tip, cycloheptatriene precursors **1** and **4** were co-deposited on a clean Au(111) surface kept at a higher temperature (325 °C) than that for the separate deposition to ensure complete planarization of **2**. Subsequently, the tip apex was terminated with a CO molecule for bond-resolved imaging (Figure 3A). We identified single molecules as well as the formation of self-assembled domains on the surface (Figure S16). Based on individual observations of HBC and 6F-HBC (Figures 1E and 2E), we assigned the single molecules to HBCs and the self-assembled molecules to 6F-HBCs, respectively. Their electronic properties were analyzed with d*I*/d*V* curves measured at the sites indicated with red and blue crosses in Figure 3A as well as with a white cross for reference. The energies of PIR and NIR for HBC were −1.37 and 2.10 V, respectively (Figure 3B). Thus, fluorine substitution causes downward shifts of both PIR and NIR levels by approximately 0.1 and 0.3 V, respectively. The observed fluorination effect on the electronic property was also reported in perfluoropetacene^36,37^ and was further confirmed by our density functional theory calculation at the B3LYP/6-311+G­(d,p) level (Figure S17). The calculated electrostatic potential map of HBC (Figure S18) indicates that the positive values on the outer regions are responsible for the repulsion between HBCs. In contrast, the presence of both positive and negative values in 6F-HBC suggests that the self-assembly is caused by hydrogen bonding, as observed in Figure 3A. To investigate the spatial distributions of HBC and 6F-HBC, constant-height d*I*/d*V* maps were recorded at the corresponding energies (Figure 3C and D). Although the energy levels of NIR and PIR were shifted, the contrast of the molecular orbitals were almost identical. Note that since the measurements were performed using a CO-terminated tip, the spatial resolution of the d*I*/d*V* maps was significantly increased due to the effect of the p_*z*_ CO orbital.^38^ Taking advantage of the coexistence of HBC and 6F-HBC in the same scan area, we investigated the detailed contrast of the corresponding bond-resolved image using the same CO-terminated tip (Figure 3E and F). A significant difference in the distortion of the outer phenyl rings was identified, as indicated by the blue arrows. The non-fluorinated phenyl moieties appear wider than the fluorinated ones. These different distortions of the phenyls result from different distributions of the total charge density,^39^ caused by fluorine substitution. Furthermore, *p*-fluorophenyl fragments exhibit a faint feature pointing outward, which corresponds to the C–F bond. Based on these features, careful inspection of the fluorophenyls revealed that several 6F-HBCs located at the edge of the molecular film incorporated a non-fluorinated phenyl group, which is likely synthesized by hydrodefluorination (Figure S19).

**Figure 3:**
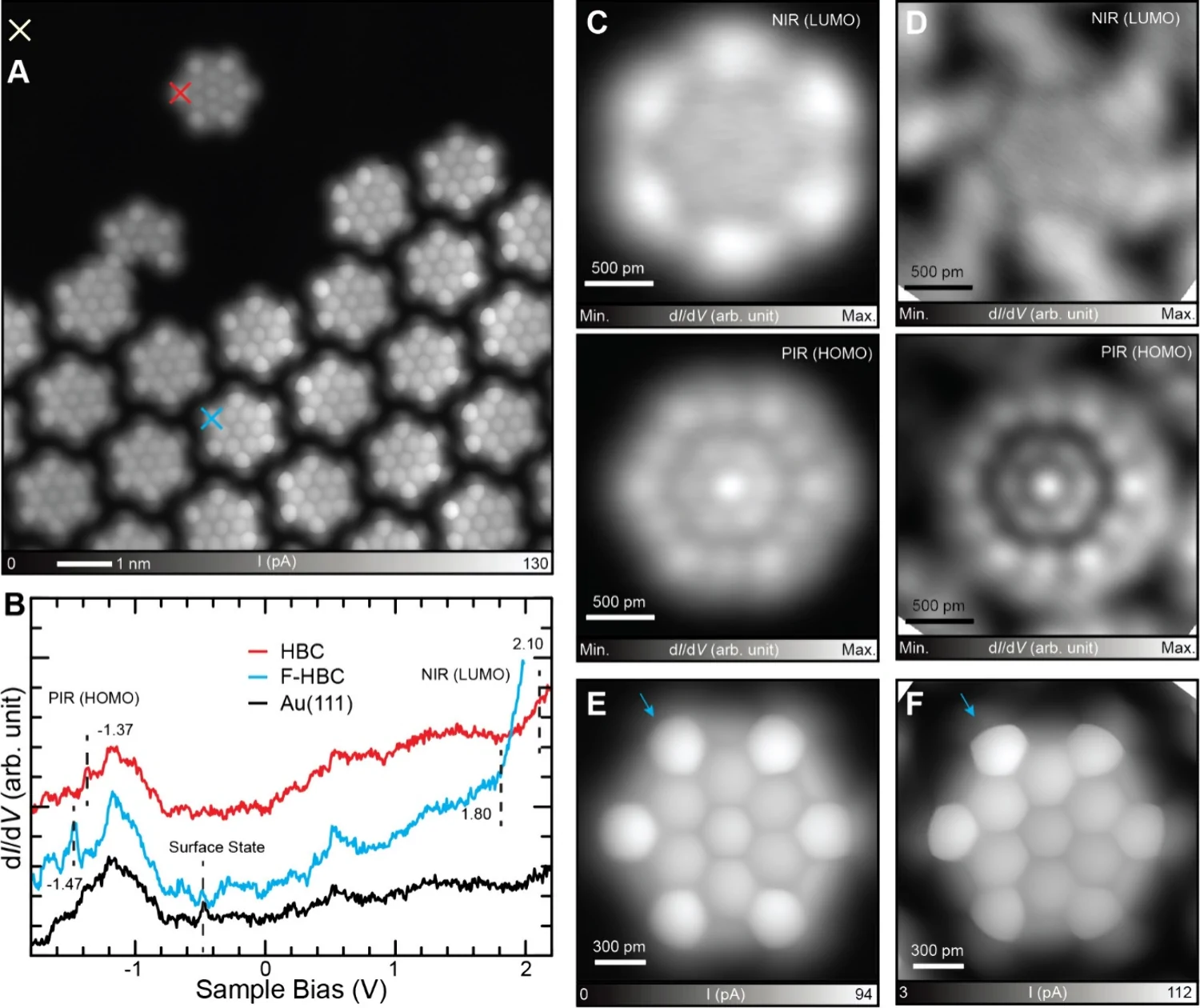
Comparison of HBC and 6F-HBC using a CO-terminated tip. (A) Constant-height bond-resolved image taken after depositing **1** and **4** on a clean Au(111) surface kept at 325 °C. The products marked with red and blue crosses correspond to HBC and 6F-HBC, respectively. (B) d*I*/d*V* curves (red and blue) measured at the sites indicated by the red and blue crosses in panel A, respectively. The black curve was measured at the site indicated by the white cross as a reference. (C) Constant-height d*I*/d*V* maps of HBC measured at 2.10 V (NIR) and −1.37 V (PIR). (D) Constant-height d*I*/d*V* maps of 6F-HBC measured at 1.80 V (NIR) and −1.47 V (PIR). Bond-resolved images of individual (E) HBC and (F) 6F-HBC taken with the same CO-terminated tip. Arrows indicate the different distortions of the outer phenyl rings. Scanning parameters: (A) *V* = 5 mV. (B–D) V_ac_ = 10 mV. (E and F) *V* = 4 mV.

In summary, we successfully synthesized HBC and 6F-HBC from heptaphenylcycloheptatriene (**1**) and hepta­(*p*-fluorophenyl)­cycloheptatriene (**4**), respectively, via dissociation of phenylcarbenic fragments followed by cyclodehydrogenation on a Au(111) surface. Their chemical structures and electronic properties were characterized by using high-resolution STM and STS. While HBC was obtained through the stepwise thermal synthesis of hexaphenylbenzene, the fluorinated analogue was desorbed from the surface upon annealing at 250 °C. We discovered that depositing hepta­(*p*-fluorophenyl)­cycloheptatriene on a clean Au(111) surface kept at 300 °C yielded 6F-HBC. To investigate both HBC and 6F-HBC with the same CO-terminated tip, both precursors were co-deposited on a hot Au(111) substrate. We observed that the fluorine substitution lowers the NIR and PIR levels by 0.1 and 0.3 V, respectively. However, the spatial distribution of both orbitals of HBC are almost identical to that of 6F-HBC. In contrast, fluorination modulates the total charge density at the outer phenyls, resulting in distinct contrast in the bond-resolved images. These findings highlight the potential of the retro-Büchner reaction as well as the role of the cycloheptatriene core and fluorine substitution for on-surface synthesis.

## Supplementary Material



## Notes

Synthesis and characterization (^1^H, ^19^F, and ^13^C NMR and mass spectrometry) of hepta­(*p*-fluorophenyl)­cycloheptatriene **4** and its precursors, methods including details of STM experiments, additional STM images, and DFT calculations of HBC (**3**) and 6F-HBC (**6**) (DOCX)
