# Supplementary material for: On-Surface Synthesis of Hexabenzocoronenes via a Retro-Büchner Reaction of Heptaarylcycloheptatrienes
Source: J Phys Chem Lett. 2026 Aug 25;17(42):12171–8. doi: 10.1021/acs.jpclett.6c01675 (PMC13700090; doi:10.1021/acs.jpclett.6c01675)
Supplement: Supplementary file 1 [file jz6c01675_si_001.docx]

*Supplementary Informations for*

On-Surface Synthesis of Hexabenzocoronenes *via* Retro-Büchner Reaction of Heptaarylcycloheptatrienes

Shigeki Kawai^1,2^*, Haruka Tsuji^1^, Donglin Li^1^, Pranshu Pal^3^, Oscar Custance^1^, Toshikaze Kariyado^4^, Yorie Ohno^5^, Yohei Hattori^5^, Thomas A. Hector^6^, Claire Kammerer^6^, Gwénaël Rapenne^5,6^*

^1^Center for Basic Research on Materials, National Institute for Materials Science, 1-2-1 Sengen, Tsukuba, Ibaraki 305-0047, Japan.

^2^*Graduate School of Pure and Applied Sciences, University of Tsukuba, Tsukuba, Ibaraki 305-8571, Japan.*

*^3^Department of Metallurgical and Materials Engineering, National Institute of Technology Srinagar, Hazratbal, Srinagar, Jammu and Kashmir 190006, India.*

^4^Research Center for Materials Nanoarchitectonics (MANA), National Institute for Materials Science, 1-1 Namiki, Tsukuba, Ibaraki 305-0044, Japan.

^5^*Division of Materials Science, Nara Institute of Science and Technology, 8916-5 Takayama, Ikoma, 630-0192, Japan.*

^6^*Univ Toulouse, CNRS, CEMES, 29 rue Marvig, 31055 Toulouse, France.*

**SUMMARY**

**1.** Synthetic procedures

**a.** General synthetic methods and materials	page S3

**b.** Tris(4-fluorophenyl)cyclopropenylium hydrogen dichloride	page S3

**c.** Tris(4-fluorophenyl)cyclopropene	page S4

**d.** Hepta(4-fluorophenyl)cycloheptatriene (**4**)	page S5

**2.** NMR and mass spectra

**a.** Tris(4-fluorophenyl)cyclopropenylium hydrogen dichloride	page S6

**b.** Tris(4-fluorophenyl)cyclopropene	page S7

**c.** Hepta(4-fluorophenyl)cycloheptatriene (**4**)	page S9

**3.** STM experiments and DFT calculations	page S12

**5.** References	page S21

**Methods**

**1. Synthetic procedures**

**a.** General synthetic methods and materials

All reagents and solvents were used without further purification, unless otherwise stated. Anhydrous benzene, ethyl acetate, and hexane were purchased from Fujifilm Wako Pure Chemical Corporation. Potassium *tert*-butoxide, 1M HCl solution in Et_2_O, NaBH_4_, and *p*-xylene were purchased from Tokyo Chemical Industry Co., Ltd. EtOH, CH₂Cl₂, Et₂O, and sodium sulfate were purchased from Nacalai Tesque, Inc. 1-(Dichloromethyl)-4-fluorobenzene was purchased from BLD Pharmatech Ltd. CDCl₃ was purchased from Eurisotop. 1,2-Bis(4-fluorophenyl)ethyne,^[S1]^ tetrakis(4-fluorophenyl)cyclopentadienone^[S2]^ and heptaphenylcyclo-heptatriene^[S3]^ (**1**) were prepared according to literature procedures. Silica gel column chromatography and thin-layer (TLC) chromatography were performed using CHROMATOREX PSQ 100B and Merck silica gel 60 (F254) TLC plates, respectively. ^1^H and ^13^C NMR spectra were recorded on a JEOL JNM-ECX400P (400 MHz for ^1^H; 151 MHz for ^13^C and ^19^F-NMR spectra were recorded on a JEOL JNM-ECX500 (470 MHz) at a constant temperature of 293 K unless otherwise specified. Tetramethylsilane (TMS) was used as an internal reference for ^1^H and ^13^C NMR measurements in CDCl₃. A residual solvent peak was used as an internal reference for ^1^H NMR measurements in CDCl₃. Chemical shifts (δ) are reported in ppm. Coupling constants (*J*) are given in Hz and the following abbreviations have been used to describe the signals: singlet (s); broad singlet (br. s); doublet (d); triplet (t); doublet of doublet (dd); multiplet (m). EI mass spectrometry was performed using JEOL JMS-700. MALDI-TOF mass spectrometry was performed using JEOL JMS-S300 spectrometer. Elemental analysis was conducted using Perkin-Elmer 2400 II CHNS/O. High-resolution mass spectrometry was conducted using JEOL JMS S-3000 MALDI-TOF MS spectrometer.

**b.** Tris(4-fluorophenyl)cyclopropenylium hydrogen dichloride

Anhydrous benzene (15.5 mL) was added to 1,2-bis(4-fluorophenyl)ethyne^[S1]^ (1.71 g, 8.00 mmol) and potassium *tert*-butoxide (2.10 g, 18.7 mmol) in an dried up two-necked 50 mL round-bottomed flask fitted with a reflux condenser and a septum under a nitrogen atmosphere. 1-(dichloromethyl)-4-fluorobenzene (1.6 mL, 11.9 mmol) was added dropwise over a period of 5 min and the reaction mixture was then heated to reflux for 3h. After cooling, water (26 mL) was added to dissolve the inorganic salts. The layers were separated and the aqueous layer extracted with Et_2_O (2 x 30 mL). The combined organic extracts were dried over Na_2_SO_4_, filtered, and the solvent was evaporated to give a brown residue. The residue was dissolved in CH_2_Cl_2_ (6 mL), cooled in an ice-water bath, and *ca.* 1M HCl solution in Et_2_O (35 mL) was added. The precipitate was collected by filtration, and the solid was washed with a minimum amount of Et_2_O and dried under vacuum to yield a cream-colored powder (2.05 g, 5.19 mmol, 65%). The product was used in the next step without further purification.

^1^H-NMR (400 MHz, CD_3_CN): δ 8.61 (dd, *J* = 8.2, 5.5 Hz, 6H), 7.63 (dd, *J* = 8.7, 8.2 Hz, 6H).

HR-MS (EI, positive): m/z calculated for [C_21_H_12_F_3_]^+^ 321.0886; 321.0893 (found).

**c.** Tris(4-fluorophenyl)cyclopropene

In a 200 mL round-bottomed flask in an ice-water bath, NaBH_4_ (0.683 g, 18.1 mmol) was added to a solution of tris(4-fluorophenyl)cyclopropenylium hydrogen dichloride (1.78 g, 4.49 mmol) in EtOH (40 mL). The mixture was allowed to stir overnight at room temperature. The reaction was quenched with water (60 mL) then extracted with Et_2_O for three times (200 mL in all). The combined organic extracts were washed with water (50 mL), then brine (50 mL), and dried over Na_2_SO_4_. The solvent was evaporated, and the residue was purified by silica gel column chromatography (CH_2_Cl_2_: hexane = 3: 2) to yield a white solid (1.17 g, 3.63 mmol, 81%).

^1^H-NMR (400 MHz, CDCl_3_): δ 7.61 (ddd, *J* = 8.7, 5.5, 2.8 Hz, 4H), 7.13 (t, *J* = 8.7 Hz, 6H), 6.92 (t, *J* = 8.7 Hz, 2H), 3.21 (s, 1H).

^19^F-NMR (470 MHz, CDCl_3_): δ −114.1 (tt, *J* = 8.5, 5.3 Hz, 2F), -121.2 (tt, J = 8.8, 5.4 Hz, 1F).

^13^C-NMR (151 MHz, CDCl_3_): δ 163.0 (d, |*J*| = 250 Hz), 161.5 (d, |*J*| = 243 Hz), 139.6 (d, |*J*| = 2.9 Hz), 131.7 (d, |*J*| = 8.8 Hz), 127.0 (d, |*J*| = 7.2 Hz), 124.7 (d, |*J*| = 2.9 Hz), 116.2 (d, |*J*| = 21.7 Hz), 115.2 (d, |*J*| = 20.2 Hz), 111.1, 23.8.

Elemental analysis calcd for C_21_H_13_F_3_: C 78.25, H 4.07, N 0.00, found: C 78.27, H 3.90, N 0.03.

HR-MS (EI, positive): m/z calculated for [C_21_H_13_F_3_]^+^ 322.0964; 322.0962 (found).

**d.** Hepta(4-fluorophenyl)cycloheptatriene (**4**)

In a Schlenk tube, tris(4-fluorophenyl)cyclopropene (322 mg, 1.00 mmol) and tetrakis(4-fluorophenyl)cyclopentadienone^[S2]^ (456 mg, 1.00 mmol) were dissolved in *p*-xylene (3 mL) under an argon atmosphere, and stirred at 140 ℃ for 40 h. The reaction mixture was purified by silica gel column chromatography (AcOEt: hexane = 1: 9). The desired compound was recrystallized from AcOEt-hexane to give a white solid (591 mg, 0.787 mmol, 79%).

^1^H-NMR (400 MHz, acetone-d_6_): δ 8.09 (dd, *J* = 7.5, 5.3 Hz, 2H), 7.35-7.20 (m, 10H), 6.83 (t, *J* = 8.9 Hz, 8H), 6.48-6.38 (m, 8H), 5.32 (s, 1H).

^19^F-NMR (470 MHz, CDCl_3_): δ −117.3 (tt, *J* = 8.5, 5.3 Hz, 2F), −118.3 (tt, *J* = 8.5, 5.7 Hz, 2F), −118.6 (m, 1F), −119.5 (tt, *J* = 8.6, 5.5 Hz, 2F).

^13^C-NMR (151 MHz, acetone-d_6_): δ 163.0 (d, |*J*| = 243 Hz), 162.2 (d, |*J*| = 243 Hz), 162.1 (d, |*J*| = 246 Hz), 161.4 (d, |*J*| = 243 Hz), 143.7, 140.3, 139.2, 137.5 (d, |*J*| = 2.9 Hz), 137.3 (d, |*J*| = 3.0 Hz), 137.1, 134.4 (d, |*J*| = 8.6 Hz), 133.8 (d, |*J*| = 7.2 Hz), 132.7 (d, |*J*| = 8.8 Hz), 129.4 (d, |*J*| = 7.2 Hz), 116.1 (d, |*J*| = 20.2 Hz), 115.4 (d, |*J*| = 21.7 Hz), 115.0 (d, |*J*| = 21.7 Hz), 113.9 (d, |*J*| = 20.2 Hz), 57.9.

Elemental analysis calcd for C_49_H_29_F_7_: C 78.39, H 3.89, N 0.00, found: C 78.43, H 4.07, N 0.09.

HR-MS (MALDI-TOF-MS, positive): m/z calculated for [C_49_H_29_F_7_]^+^ 750.21520; 750.21535 (found).

**2. NMR and Mass spectra**

**a.** Tris(4-fluorophenyl)cyclopropenylium hydrogen dichloride

**Figure S1.** ^1^H-NMR spectrum (400 MHz, CD_3_CN) of tris(4-fluorophenyl)cyclopropenylium hydrogen dichloride.

**Figure S2.** EI mass spectrum (positive mode) of tris(4-fluorophenyl)cyclopropenylium hydrogen dichloride.

**b.** Tris(4-fluorophenyl)cyclopropene

**Figure S3.** ^1^H-NMR spectrum (400 MHz, CDCl_3_) of tris(4-fluorophenyl)cyclopropene.

**Figure S4.** ^13^C-NMR spectrum (151 MHz, CDCl_3_) of tris(4-fluorophenyl)cyclopropene.

**Figure S5.** ^19^F-NMR spectrum (470 MHz, CDCl_3_) of tris(4-fluorophenyl)cyclopropene.

**Figure S6.** EI mass spectrum (positive mode) of tris(4-fluorophenyl)cyclopropene.

**c.** Hepta(4-fluorophenyl)cycloheptatriene (**4**)

**Figure S7.** ^1^H-NMR spectrum (400 MHz, acetone-d_6_) of hepta(4-fluorophenyl)cycloheptatriene.

**Figure S8.** ^13^C-NMR spectrum (151 MHz, acetone-d_6_) of hepta(4-fluorophenyl)cycloheptatriene.

**Figure S9.** ^19^F-NMR spectrum (470 MHz, CDCl_3_) of hepta(4-fluorophenyl)cycloheptatriene.

**Figure S10.** HR-MALDI-TOF mass spectrum of hepta(4-fluorophenyl)cycloheptatriene (positive mode), full spectrum (bottom) and isotopic pattern of the molecular peak.

**3. STM experiments**

A homemade low-temperature scanning tunneling microscope (STM)/atomic force microscopy system equipped with a specially designed UNISOKU microscope head was used, operating at 4.5 K under ultrahigh vacuum conditions (P < 3×10^-10^ mbar). The Au(111) substrates were cleaned through multiple cycles of sputtering with Ar^+^ ions and annealing at 500 °C for 15 minutes. The sample temperature was monitored using a thermocouple. Heptaphenylcycloheptatriene (**1)** and hepta(*p*-fluorophenyl)cycloheptatriene (**4**) as precursors were deposited on clean Au(111) surfaces. Knudsen cells were used to evaporate precursor molecules. The sublimation temperatures of **1** and **4** were 160 °C and 150 °C, respectively. STM images were obtained with a chemically etched tungsten tip. Bond-resolved STM (BR-STM) images were conducted using a CO-functionalized tip in constant-height mode.

**Figure S11.** Large-scale STM topography of as-deposited heptaphenylcycloheptatriene (**1**) with a narrow Z scale contrast. The herringbone structure of the Au(111) substrate can be seen on the molecular file as indicated by blue lines. Scanning parameter: *V* = 0.2 V and *I* = 5 pA.

**Figure S12.** Defect of hexabenzocoronene (HBC, **3**). (**A**) Large-scale constant-height bond-resolved image, showing individual HBCs and fused HBCs. (**B**) Dimerized HBC. (**C**) HBC with an additional benzene ring. (**D**-**F**) Randomly fused HBC and defected HBCs. Scanning parameter: (A) *V* = 10 mV. (B) *V* = 10 mV. (C) *V* = 4 mV. (D) *V* = 5 mV. (E) *V* = 10 mV. (F) *V* = 10 mV.

**Figure S13.** (**A**) Close-up view of Figure 2D to clarify the tilt direction of the phenyl units, and (**B**) the corresponding image with superimposed arrows. The green and blue arrows indicate the clockwise and anticlockwise directions of the title, respectively.

**Figure S14.** Defects of 6F-HBCs. (**A**) 6F-HBC with an additional benzene ring. (**B**) One phenyl ring was eliminated before the cyclodehydrogenation. Scanning parameter: (A, B) *V* = 5 mV.

**Figure S15.** Bond-resolved image of the self-assembled 6F-HBCs. The defects (five on this image) were indicated by blue arrows. Scanning parameter: *V* = 5 mV. Number of image pixels: 2048 × 2048.

**Figure S16.** (**A**) Large-scale STM topography of HBC and 6F-HBC synthesized by co-deposition of precursors **1** and **4** on a Au(111) surface kept at 300 °C. (**B**-**D**) Bond-resolved images taken at the areas indicated by blue (**B**), red (**C**) and green (**D**) squares in **A**. Scanning parameter: (A) *V* = 100 mV and *I* = 50 pA. (B,C,D) *V* = 5 mV.

**Figure S17.** Energy levels and electron density distributions of the frontier orbitals of **HBC** and **6F-HBC**. The theoretical calculations were performed at the B3LYP/6-311+G(d,p) level of theory with the Gaussian 16^[S4]^ package.

**Figure S18.** Chemical structures (top) and their electrostatic potential surfaces (bottom) of **HBC** and **6F-HBC** (Isovalue for density: 0.001).

**Figure S19.** Hydrodefluorination of 6F-HBC. (**A**) Bond-resolved image taken around the edge of the self-assembled molecular film. (**B**) Superimposed chemical structure, indicating the hydrodefluorination sites by blue arrows. The C-F bond points towards the H-C bond of the neighboring 6F-HBC, indicating that the self-assembled structure is condensed by the hydrogen bonding. Scanning parameter: *V* = 4 mV.

**Table S1.** Cartesian coordinates (Å) of **HBC** at the B3LYP/6-311+G(d,p) level of theory. The calculated structure shows a small remaining imaginary frequency (f = −3.80 cm^−1^).

| C  C  C  C  C  C  C  C  C  C  C  C  C  C  C  C  C  C  C  C  C  C  C  C  C  C  C  C  C  C  C  C  C  C  C  C  C  C  C  C  C  C  H  H  H  H  H  H  H  H  H  H  H  H  H  H  H  H  H  H | -0.24189400  -1.33372100  -1.09173700  0.24189200  1.33371800  1.09174000  -0.48775400  2.20199800  2.69044000  0.48774400  -2.20197000  -2.69045900  0.60005400  0.33614900  -0.96240800  -2.02765800  -1.81920500  2.94032700  4.26205700  5.32457900  5.09006800  3.78738000  -3.53806400  -4.59142100  -4.35321000  -3.05632300  -1.96728300  -0.60005500  -0.33619500  0.96235200  2.02762200  1.81917700  3.53810300  4.59154500  4.35340100  3.05647900  1.96733600  -2.94037800  -4.26218200  -5.32471200  -5.09013800  -3.78739100  1.14869700  -1.14511800  -3.03015700  4.47308800  6.34039500  5.93878400  -5.61373200  -5.18237300  -2.89798900  -1.14879000  1.14503800  3.03012600  5.61389100  5.18264400  2.89825400  -4.47331700  -6.34058700  -5.93885400 | 1.39985900  0.49055600  -0.90947400  -1.39983400  -0.49053800  0.90949300  2.82356400  1.83508200  -0.98997900  -2.82353100  -1.83508400  0.98996400  3.73898800  5.10770700  5.58169500  4.69946100  3.32095800  -2.38972300  -2.84884000  -1.96418200  -0.59963500  -0.08697000  -1.35228300  -2.27216300  -3.63621000  -4.11350600  -3.23771000  -3.73894400  -5.10758200  -5.58151900  -4.69933900  -3.32092300  1.35224900  2.27211300  3.63617100  4.11348200  3.23771500  2.38969800  2.84873500  1.96403600  0.59950900  0.08691700  5.81730800  6.64362900  5.09633100  -3.90841700  -2.33992000  0.06668300  -1.92906300  -4.33054600  -5.18215200  -5.81710100  -6.64336200  -5.09612400  1.92900400  4.33050800  5.18214600  3.90828600  2.33973000  -0.06685900 | 0.01036500  -0.00422800  -0.00674800  0.01040000  -0.00419100  -0.00676900  0.06108800  -0.03802600  -0.02338800  0.06133400  -0.03820100  -0.02323500  0.09165400  0.22718900  0.31007600  0.24185400  0.10795100  0.01613800  -0.04247800  -0.11869700  -0.13692200  -0.09232500  -0.10857900  -0.18740800  -0.19398200  -0.10579800  -0.01596500  0.09205400  0.22848400  0.31192400  0.24318700  0.10836900  -0.10818500  -0.18598600  -0.19206600  -0.10453900  -0.01564200  0.01645100  -0.04120000  -0.11676900  -0.13551500  -0.09195500  0.28702700  0.42853000  0.31074700  -0.04008800  -0.16414800  -0.18869300  -0.25186600  -0.26823600  -0.12214200  0.28874800  0.43122000  0.31255500  -0.24981500  -0.26539900  -0.12046800  -0.03835400  -0.16127900  -0.18666100 |
| --- | --- | --- | --- |

**Table S2.** Cartesian coordinates (Å) of **6F-HBC** at the B3LYP/6-311+G(d,p) level of theory.

| C  C  C  C  C  C  C  C  C  C  C  C  C  C  C  C  C  C  C  C  C  C  C  C  C  C  C  C  C  C  C  C  C  C  C  C  C  C  C  C  C  C  F  F  F  F  F  F  H  H  H  H  H  H  H  H  H  H  H  H | -1.15149900  -1.29572900  -0.14421100  1.15150200  1.29573200  0.14421300  2.32264900  3.62432100  3.77249900  2.61352600  2.76769700  1.58448500  0.29084700  -0.85664400  -2.18799500  -2.32264400  -3.62431600  -3.77249400  -2.61352400  -2.76769900  -1.58448800  -0.29084800  0.85664400  2.18799900  -3.33399400  -4.57893200  -4.74697300  1.70672000  0.57341200  -0.69475200  3.33400200  4.57893900  4.74697800  -5.04074600  -5.15237000  -4.05227600  5.04074800  5.15236800  4.05227200  -1.70672600  -0.57342200  0.69474400  0.71051600  -5.67369200  6.38419300  5.67370000  -0.71052900  -6.38419700  -3.28685000  -5.75583800  2.66429000  -1.53191800  3.28686800  5.75584300  -5.95123300  -4.22406400  5.95123800  4.22405300  -2.66429600  1.53190100 | -0.83131600  0.58156900  1.41288700  0.83132200  -0.58156400  -1.41288200  1.67682500  1.10332600  -0.34842900  -1.17304700  -2.58706000  -3.44123600  -2.84984700  -3.69038200  -3.09283000  -1.67682200  -1.10332600  0.34842700  1.17304900  2.58705900  3.44123700  2.84985200  3.69038800  3.09283600  -3.89564200  -3.30577900  -1.93847000  -4.83509700  -5.61833000  -5.08021000  3.89564300  3.30577900  1.93847100  0.93949900  2.31258000  3.14179200  -0.93950900  -2.31259000  -3.14179900  4.83509600  5.61833400  5.08021700  -6.96156800  -4.09609200  -2.86546100  4.09609100  6.96157100  2.86544800  -4.97412800  -1.55433000  -5.33351600  -5.76176100  4.97412800  1.55433300  0.35946800  4.20756700  -0.35948600  -4.20757600  5.33351400  5.76177600 | -0.00002500  0.00001600  0.00002800  -0.00003400  0.00001800  0.00004500  -0.00018600  -0.00036500  -0.00016700  0.00003500  0.00024400  0.00035300  0.00016300  0.00012600  -0.00017100  -0.00015400  -0.00032000  -0.00019400  0.00001600  0.00020100  0.00036500  0.00015100  0.00013500  -0.00022500  -0.00043900  -0.00065900  -0.00057600  0.00062300  0.00068200  0.00042200  -0.00056800  -0.00083000  -0.00069800  -0.00030900  -0.00013900  0.00013800  -0.00019100  0.00003700  0.00026800  0.00074300  0.00086400  0.00054100  0.00103400  -0.00098600  0.00007800  -0.00124700  0.00134000  -0.00019700  -0.00051400  -0.00071700  0.00081000  0.00051100  -0.00067400  -0.00086700  -0.00050700  0.00024400  -0.00035700  0.00041400  0.00098000  0.00067700 |
| --- | --- | --- | --- |

**5. References:**

[S1] L. E. Sattler, G. Hilt, *Chem. Eur. J.* **2021**, *27*, 605−608.

[S2] Y. Kikuzawa, T. Mori, H. Takeuchi, *Org. Lett.* **2007**, *9*, 4817−4820.]

[S3] P. K. Saha, A. Mallick, A. T. Turley, A. N. Bismillah, A. Danos, A. P. Monkman, A.-J. Avestro, D. S. Yufit, P. R. McGonigal. *Nat. Chem.* **2023**, *15*, 516−525.

[S4]	Gaussian 16, Revision C.01, M. J. Frisch, G. W. Trucks, H. B. Schlegel, G. E. Scuseria, M. A. Robb, J. R. Cheeseman, G. Scalmani, V. Barone, G. A. Petersson, H. Nakatsuji, X. Li, M. Caricato, A. V. Marenich, J. Bloino, B. G. Janesko, R. Gomperts, B. Mennucci, H. P. Hratchian, J. V. Ortiz, A. F. Izmaylov, J. L. Sonnenberg, D. Williams-Young, F. Ding, F. Lipparini, F. Egidi, J. Goings, B. Peng, A. Petrone, T. Henderson, D. Ranasinghe, V. G. Zakrzewski, J. Gao, N. Rega, G. Zheng, W. Liang, M. Hada, M. Ehara, K. Toyota, R. Fukuda, J. Hasegawa, M. Ishida, T. Nakajima, Y. Honda, O. Kitao, H. Nakai, T. Vreven, K. Throssell, J. A. Montgomery, Jr., J. E. Peralta, F. Ogliaro, M. J. Bearpark, J. J. Heyd, E. N. Brothers, K. N. Kudin, V. N. Staroverov, T. A. Keith, R. Kobayashi, J. Normand, K. Raghavachari, A. P. Rendell, J. C. Burant, S. S. Iyengar, J. Tomasi, M. Cossi, J. M. Millam, M. Klene, C. Adamo, R. Cammi, J. W. Ochterski, R. L. Martin, K. Morokuma, O. Farkas, J. B. Foresman, and D. J. Fox, Gaussian, Inc., Wallingford CT, 2016.
